# Uric Acid Induces Hepatocytes Ferroptosis Through HIF-2α/DMT1-Mediated Iron Overload

**DOI:** 10.3390/ijms27062833

**Published:** 2026-03-20

**Authors:** Tao Wang, Wanbao Zheng, Meimei Guo, Jun Cao, Li Wang, Marco Sim Kah How, Youzhi Xu, Wenjie Lu

**Affiliations:** 1 School of Pharmacy, Anhui Medical University, Hefei 230032, China; 2345010146@stu.ahmu.edu.cn (T.W.); 2345010176@stu.ahmu.edu.cn (W.Z.); caojun20201314@163.com (J.C.); 2 Basic Medical College, Anhui Medical University, Hefei 230032, China; 2345010085@stu.ahmu.edu.cn (M.G.); 2345010251@stu.ahmu.edu.cn (L.W.); 3 International College, Anhui Medical University, Hefei 230032, China; marcosim662001@gmail.com

**Keywords:** hyperuricemia, ferroptosis, HIF-2α, DMT1, liver injury, lipid peroxidation

## Abstract

Hyperuricemia is associated with liver dysfunction, yet its molecular mechanisms remain unclear. This study investigated high uric acid (HUA)-induced hepatocyte injury using a hyperuricemia mouse model (HUM) and uric acid (UA)-treated L02 cells. HUM exhibited elevated aspartate aminotransferase (AST)/alanine aminotransferase (ALT) and pathological liver changes. Transmission electron microscopy (TEM) confirmed ferroptotic hallmarks, including mitochondrial shrinkage and increased membrane density. UA exposure upregulated NADPH oxidase 4 (NOX4), increased reactive oxygen species (ROS), and promoted lipid peroxidation (LPO), accompanied by intracellular Fe^2+^ accumulation. Mechanistically, UA increased hypoxia-inducible factor-2α (HIF-2α) expression, subsequently upregulating iron transporters divalent metal transporter 1 (DMT1) and transferrin receptor (TFRC). Deferoxamine (DFO) treatment effectively reversed Fe^2+^ overload and alleviated oxidative stress. Notably, pharmacological inhibition or genetic knockdown of HIF-2α specifically suppressed DMT1 upregulation and restored iron homeostasis, while TFRC expression remained unaffected. Blocking the HIF-2α/DMT1 axis significantly reduced LPO and mitochondrial dysfunction. These findings demonstrate that HUA induces hepatocyte ferroptosis through HIF-2α-mediated DMT1 upregulation, leading to Fe^2+^ overload and mitochondrial impairment. This study identifies the HIF-2α/DMT1 pathway as a key driver of HUA-induced liver injury and a potential therapeutic target.

## 1. Introduction

Uric acid (UA) is the final product of purine metabolism in the human body, and impaired purine metabolism can lead to elevated serum uric acid levels, ultimately resulting in hyperuricemia [1]. Clinically, hyperuricemia is defined as serum uric acid >6.0 mg/dL in women and >7.0 mg/dL in men [2]. Hyperuricemia has gradually become an important global public health issue [3]. The prevalence of hyperuricemia among Chinese adults has increased significantly in recent years, rising from 11.1% in 2015–2016 to 14.0% in 2018–2019 [4]. Clinical studies have found that uric acid participates in the development of multiple diseases, including metabolic syndrome, diabetes, non-alcoholic fatty liver disease, coronary heart disease, heart failure, and chronic kidney disease [5]. Xanthine oxidase catalyzes the hydroxylation of hypoxanthine to generate xanthine and the hydroxylation of xanthine to generate uric acid. Since the liver has the highest protein expression of xanthine oxidase, hepatocytes are constantly exposed to very high levels of uric acid [6]. Elevated serum uric acid is a potential risk factor for liver dysfunction [7,8]. Studies have shown that patients with hyperuricemia are twice as likely to have abnormal levels of liver function markers aspartate aminotransferase (AST) and alanine aminotransferase (ALT) compared to normal individuals [9]. Furthermore, elevated uric acid levels increase the risk of liver cirrhosis and its related severity [10]. Similarly, another study demonstrated that elevated uric acid levels are associated with increased incidence and disease severity of non-alcoholic fatty liver disease [11]. However, the molecular mechanisms by which high uric acid causes liver injury have not been fully elucidated.

Given that hepatocytes are uniquely vulnerable to iron-mediated oxidative injury due to their central role in iron metabolism, ferroptosis—an iron-regulated form of regulated necrotic cell death—represents a compelling mechanistic candidate underlying high uric acid-induced liver injury [12]. Iron is essential for cell growth and division, controlling heme and iron-protein formation, and participating in oxygen transport, energy metabolism, neurotransmitter synthesis, DNA synthesis, and other processes. However, iron concentration must be strictly controlled as iron participates in free radical generation, causing damage to biomolecules and promoting oxidative stress. Iron metabolism dysregulation, particularly excessive iron acquisition and retention, can induce tumor and cancer growth [13]. Iron is absorbed from the daily diet into duodenal villus epithelial cells. After transferrin (TF) carrying ferric ions (Fe^3+^) binds to the transferrin receptor (TFRC) on the plasma membrane, the plasma membrane forms endosomes that internalize TF carrying Fe^3+^ into the cell. The acidic environment in the endosome promotes the release of Fe^3+^ from TF. Before being released into the cytoplasm through divalent metal transporter 1 (DMT1), Fe^3+^ in the endosome is reduced to ferrous ions (Fe^2+^) by six-transmembrane epithelial antigen of prostate 3 (STEAP3) [14,15]. Fe^2+^ is then transported for functional use or stored in the labile iron pool (LIP) and ferritin. Ferroptosis is accompanied by elevated intracellular free Fe^2+^, reactive oxygen species (ROS), and lipid peroxides. The highly oxidative Fe^2+^ readily undergoes the Fenton reaction with H_2_O_2_, generating hydroxyl radicals that directly attack lipids, damaging cell membranes and causing cell death. Increasing evidence shows ferroptosis is associated with ischemia–reperfusion injury, Alzheimer’s disease, Parkinson’s disease, stroke, and cancer [16].

Hypoxia-inducible factor (HIF) is a heterodimer with three α subunit isoforms: HIF-1α, HIF-2α, and HIF-3α [17]. These subunits are modified by iron-dependent prolyl hydroxylases (PHDs) and then degraded through interaction with the ubiquitin ligase von Hippel-Lindau (VHL). Under hypoxia or iron chelation, PHD activity is inhibited, allowing these subunits to bind with HIF-1β to form heterodimers. The heterodimeric HIF binds to hypoxia response elements (HREs) in DNA sequences to activate transcription of genes encoding secreted growth and pro-angiogenic factors, participating in the regulation of metabolism, angiogenesis, apoptosis, and cellular stress [18]. Studies have shown that ferroportin (FPN), hepcidin, and erythropoietin (EPO), under the control of the VHL/HIF pathway, can coordinately regulate iron metabolism and oxygen transport, such as providing iron for erythropoiesis [19]. HIF-2α is a key regulator of DMT1 [20]. Under systemic iron deficiency and erythropoietic demands in systemic hypoxia, HIF-2α can adaptively increase and directly activate iron absorption mechanisms through transcription. Therefore, HIF-2α is both necessary and sufficient for mediating iron absorption [21,22,23,24]. Therefore, the present study aimed to investigate whether hyperuricemia activates the HIF-2α/DMT1 signaling axis to drive intracellular iron overload and ferroptotic cell death in hepatocytes, and to evaluate HIF-2α as a potential therapeutic target for hyperuricemia-associated liver injury, using both a hyperuricemia mouse model (HUM) and UA-treated L02 hepatocytes.

## 2. Results

### 2.1. Hyperuricemia Induces Ferroptotic Cell Death in Hepatocytes

**Figure 1:**
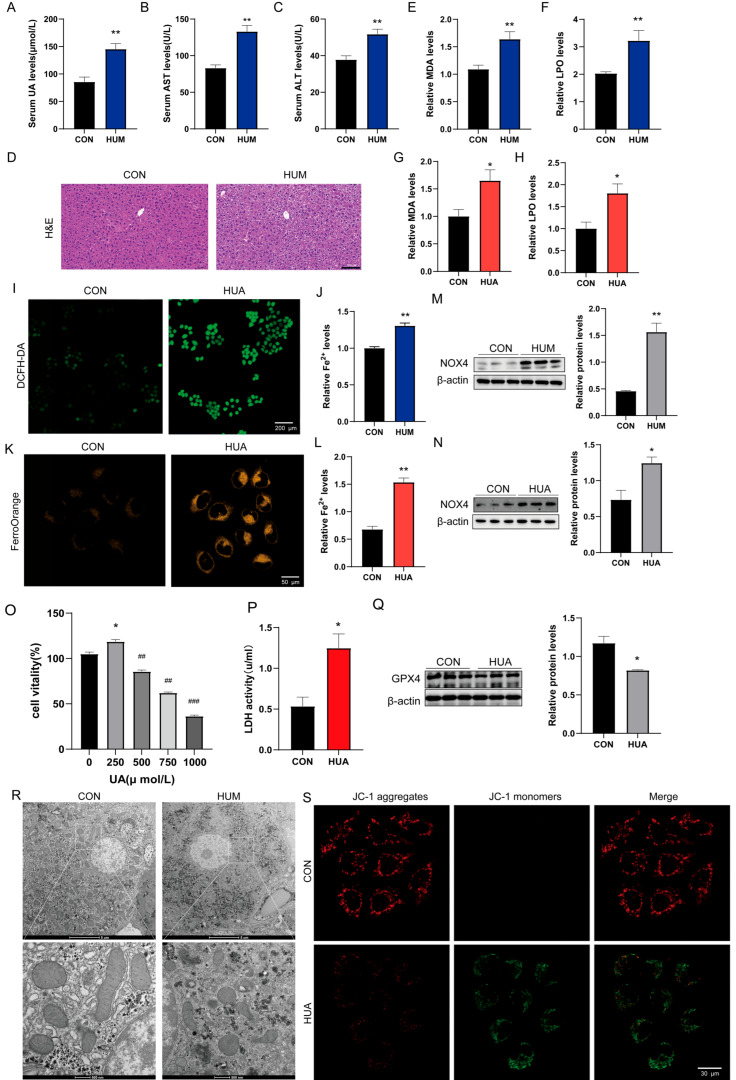
Hyperuricemia induces ferroptosis in hepatocytes. (**A**–**C**) Serum levels of uric acid (UA), aspartate aminotransferase (AST), and alanine aminotransferase (ALT) in mice (*n* = 6). (**D**) HE staining of mouse liver tissues (*n* = 6). (**E**–**H**) Malondialdehyde (MDA) and lipid peroxidation (LPO) levels in mouse liver (*n* = 6) and L02 hepatocytes (*n* = 3). (**I**) DCFH-DA probe fluorescence in L02 cells (*n* = 3). (**J**–**L**) Fe^2+^ levels in mouse liver (*n* = 6) and L02 cells (*n* = 3). (**M**,**N**) Western blot analysis of NOX4 protein expression in mouse liver (*n* = 6) and L02 cells (*n* = 3). (**O**) CCK8 assay for L02 cell viability (0, 250, 500, 750, 1000 μM). (**P**) LDH activity in L02 cells treated with 750 μM uric acid for 48 h (*n* = 3). (**Q**) Western blot analysis of GPX4 protein expression in L02 cells (*n* = 3). (**R**) Transmission electron microscopy (TEM) images showing mitochondrial ultrastructure in mouse liver cells. (**S**) Mitochondrial membrane potential (MMP) in L02 cells detected by JC-1 probe (*n* = 3). * *p* < 0.05, ** *p* < 0.01 vs. control group, ^##^ *p* < 0.01, ^###^ *p* < 0.001 vs. 250 µM group.

We first established the HUA mouse model and assessed hepatic function. Compared with the control group, HUA mice showed markedly elevated serum levels of UA, aspartate aminotransferase (AST), and alanine aminotransferase (ALT) (Figure 1A–C). H&E staining further revealed extensive vacuolar degeneration and disrupted hepatic architecture (Figure 1D), confirming that hyperuricemia induced substantial liver injury.

L02 cells were exposed to UA at 0, 250, 500, 750, and 1000 μM for 48 h. Cellular metabolic activity was measured using the Cell Counting Kit-8 (CCK-8) assay. UA reduced metabolic activity in a concentration-dependent manner. At 750 μM, metabolic activity was approximately 50% of the control level (Figure 1O). At 250 μM, metabolic activity was slightly higher than that of the control group. No reduction was observed at this concentration. In contrast, metabolic activity declined progressively at concentrations of 500 μM and above. To determine whether these metabolic changes were accompanied by cell injury, lactate dehydrogenase (LDH) release was measured in the culture medium. UA treatment at 750 μM for 48 h caused an approximately twofold increase in extracellular LDH activity compared with control (Figure 1P). Increased LDH release indicates loss of membrane integrity. Based on these results, 750 μM UA was used as the working concentration for subsequent mechanistic experiments.

What mechanisms underlie this hepatotoxicity? We turned our attention to oxidative stress, a well-established contributor to liver pathology. Both malondialdehyde (MDA) and Lipid peroxidation (LPO) were significantly increased in HUA mouse livers and UA-exposed L02 hepatocytes (Figure 1E–H). DCFH-DA probe fluorescence was elevated in UA-treated L02 cells (Figure 1I). It should be noted that DCFH-DA can be oxidized directly by Fe^2+^ via 1-electron reactions; therefore, the observed DCF fluorescence may partly reflect iron-mediated oxidation rather than exclusively non-metal ROS. However, the conclusion of oxidative stress is robustly supported by multiple iron-independent markers described below.

Given that lipid peroxidation frequently accompanies iron dyshomeostasis, we quantified intracellular Fe^2+^ content. Both in vivo and in vitro models showed elevated Fe^2+^ levels (Figure 1J–L), indicating iron overload. We next examined NOX4 as a potential mediator and found it upregulated in hyperuricemic livers and UA-exposed L02 cells (Figure 1M,N). Most critically, glutathione peroxidase 4 (GPX4) expression was significantly suppressed in UA-treated cells (Figure 1Q). GPX4 is the central and most widely accepted molecular gatekeeper of ferroptosis; its downregulation is both necessary and sufficient to trigger ferroptotic cell death. The concurrent elevation of lipid peroxidation markers (MDA, LPO) with GPX4 depletion is consistent with loss of GPX4-mediated phospholipid hydroperoxide detoxification and subsequent ferroptotic execution.

The combination of lipid peroxidation, iron accumulation, NOX4 induction, and GPX4 suppression strongly suggested ferroptosis as the underlying mechanism. Ultrastructural examination by transmission electron microscopy (TEM) confirmed characteristic ferroptotic features: mitochondrial shrinkage with condensed membranes and cristae reduction (Figure 1R). Importantly, nuclear morphology remained relatively preserved, lacking the chromatin condensation, margination, and fragmentation characteristic of apoptosis—a key morphological distinction confirming ferroptosis rather than apoptotic cell death. JC-1 staining demonstrated mitochondrial membrane potential (MMP) collapse (Figure 1S), indicating irreversible mitochondrial dysfunction associated with cell death.

To definitively establish ferroptosis as the mode of cell death, we employed Ferrostatin-1 (Fer-1), the gold-standard ferroptosis-specific inhibitor. L02 cells were pretreated with Fer-1 (1 μM, 2 h) prior to UA exposure. Fer-1 treatment significantly rescued both UA-induced cell viability loss (Figure S1A) and LDH release (Figure S1B). This pharmacological rescue by a ferroptosis-specific inhibitor provides definitive evidence that cell death occurs via the ferroptotic pathway, as Fer-1 specifically inhibits lipid peroxidation and is ineffective against apoptosis, necroptosis, or other cell death modalities.

### 2.2. Hyperuricemia Upregulates HIF-2α Expression and Iron Transport Proteins

**Figure 2:**
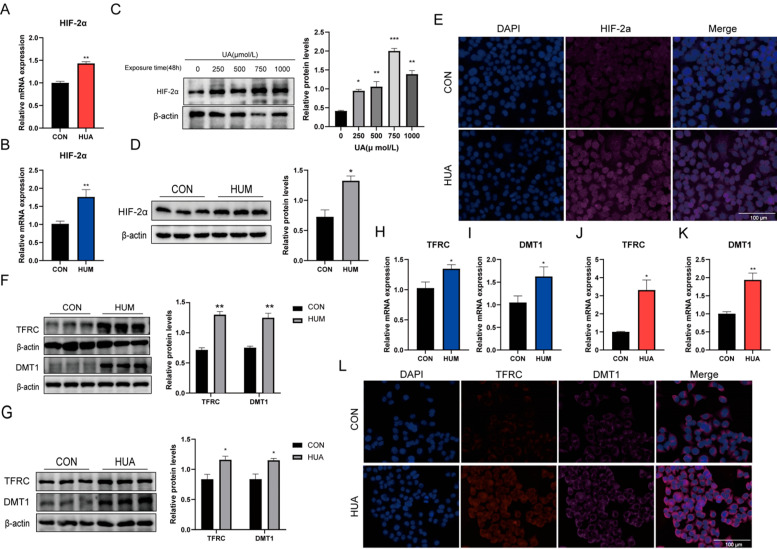
Hyperuricemia upregulates HIF-2α expression and iron transport proteins. (**A**,**B**) HIF-2α mRNA levels in L02 hepatocytes (*n* = 3) and mouse liver (*n* = 6). (**C**) HIF-2α protein expression in L02 cells treated with different UA concentrations (0, 250, 500, 750, 1000 μM) (*n* = 3). (**D**) HIF-2α protein expression in mouse liver (*n* = 6). (**E**) Immunofluorescence staining of HIF-2α in L02 cells (*n* = 3). (**F**,**G**) Protein expression of TFRC and DMT1 in mouse liver tissues (*n* = 6) and L02 hepatocytes (*n* = 3). (**H**,**I**) TFRC and DMT1 mRNA levels in mouse liver (*n* = 6). (**J**,**K**) DMT1 and TFRC mRNA levels in L02 cells (*n* = 3). (**L**) Immunofluorescence staining of DMT1 and TFRC proteins in L02 cells (*n* = 3). * *p* < 0.05, ** *p* < 0.01, *** *p* < 0.001 vs. control group.

We hypothesized that HIF-2α regulates ferroptosis in hyperuricemic liver injury. HIF-2α mRNA and protein levels rose in hyperuricemic mouse livers and UA-treated L02 hepatocytes (Figure 2A,B,D). L02 cells exposed to increasing UA concentrations (0–1000 μM) showed dose-dependent HIF-2α upregulation (Figure 2C), with nuclear accumulation confirmed by immunofluorescence (Figure 2E).

Does HIF-2α activation account for the observed iron overload? We examined two key iron importers regulated by HIFs: TFRC [25] and DMT1 [20]. Western blot analysis revealed marked upregulation of both proteins in HUA mouse livers and L02 cells (Figure 2F,G). This was paralleled by increased mRNA expression of TFRC and DMT1 in both models (Figure 2H–K), and immunofluorescence staining further localized their accumulation within L02 hepatocytes (Figure 2L). These findings suggest that HIF-2α-mediated induction of TFRC and DMT1 may drive intracellular iron accumulation, thereby facilitating ferroptotic cell death in hyperuricemia.

### 2.3. Deferoxamine Alleviates Hyperuricemia-Induced Ferroptosis in Hepatocytes

The upregulation of TFRC and DMT1 points to iron overload as a potential driver of hepatotoxicity. If this hypothesis is correct, eliminating excess iron should rescue the phenotype. To test this causality, we treated UA-exposed L02 cells with the iron chelator deferoxamine (DFO).

**Figure 3:**
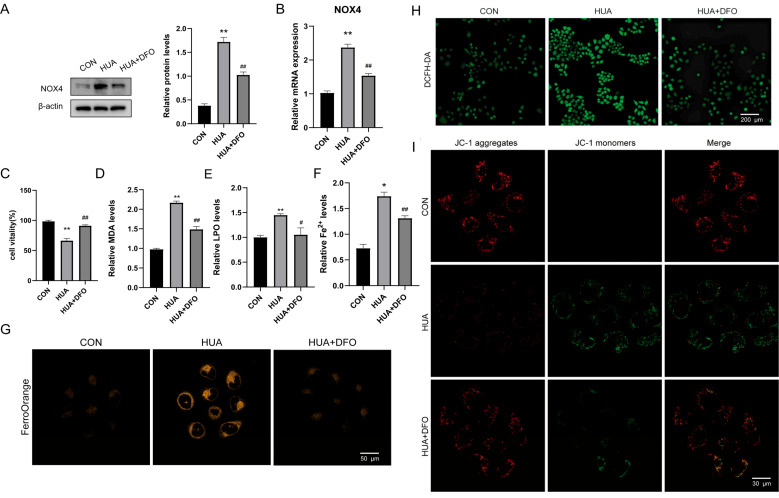
Deferoxamine alleviates hyperuricemia-induced ferroptosis in hepatocytes. (**A**) Western blot analysis of NOX4 protein expression in L02 cells after deferoxamine treatment *(n* = 3). (**B**) NOX4 mRNA levels in L02 cells after deferoxamine treatment detected (*n* = 3). (**C**) CCK-8 assay for L02 cell viability after deferoxamine treatment (*n* = 3). (**D**,**E**) MDA and LPO levels in L02 hepatocytes after deferoxamine treatment (*n* = 3). (**F**,**G**) Fe^2+^ levels in L02 cells after deferoxamine treatment (*n* = 3). (**H**) DCFH-DA probe fluorescence in L02 cells after deferoxamine treatment (*n* = 3). (**I**) MMP in L02 cells after deferoxamine treatment detected by JC-1 probe (*n* = 3). * *p* < 0.05, ** *p* < 0.01 vs. control group, ^#^ *p* < 0.05, ^##^ *p* < 0.01 vs. model group.

We first examined NOX4, the oxidative stress regulator identified earlier. DFO treatment markedly suppressed NOX4 expression at both protein and mRNA levels (Figure 3A,B), suggesting that NOX4 activation depends on iron availability. Functionally, DFO treatment significantly restored L02 cell viability that was compromised by UA exposure (Figure 3C), providing direct evidence that iron chelation protects against UA-induced cytotoxicity.

Does mitigating iron overload halt the ferroptotic cascade? The results provided a clear answer. DFO intervention effectively attenuated lipid peroxidation markers (MDA and LPO) (Figure 3D,E) and significantly reduced intracellular Fe^2+^ levels (Figure 3F,G). Consistent with these findings, DCFH-DA probe fluorescence was markedly suppressed by DFO treatment (Figure 3H), likely reflecting both reduced iron-mediated oxidation and decreased ROS generation. Crucially, this relief of oxidative stress extended to organelle function, as JC-1 staining showed a restoration of mitochondrial membrane potential (Figure 3I).

### 2.4. HIF-2α Inhibitor Alleviates Hyperuricemia-Induced Ferroptosis in Hepatocytes

Iron chelation reducing the injury was consistent with iron accumulation being functionally important, but it left open whether HIF-2α sat upstream of this process or was simply co-induced. To test that directly, we treated UA-exposed L02 cells with the HIF-2α inhibitor HIF-2α-IN-4.

**Figure 4:**
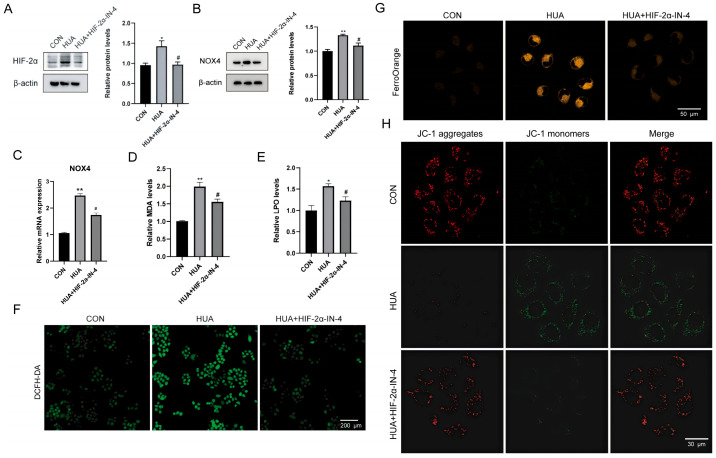
HIF-2α inhibitor alleviates hyperuricemia-induced ferroptosis in hepatocytes. (**A**,**B**) Western blot analysis of HIF-2α and NOX4 protein expression in L02 cells after HIF-2α-IN-4 treatment (*n* = 3). (**C**) NOX4 mRNA levels in L02 cells after HIF-2α-IN-4 treatment (*n* = 3). (**D**,**E**) MDA and LPO levels in L02 hepatocytes after HIF-2α-IN-4 treatment (*n* = 3). (**F**) DCFH-DA probe fluorescence in L02 cells after HIF-2α-IN-4 treatment (*n* = 3). (**G**) Fe^2+^ levels in L02 cells after HIF-2α-IN-4 treatment detected by FerroOrange probe (*n* = 3). (**H**) MMP in L02 cells after HIF-2α-IN-4 treatment detected by JC-1 probe (*n* = 3). * *p* < 0.05, ** *p* < 0.01 vs. control group, ^#^ *p* < 0.05 vs. model group.

Efficacy validation confirmed that the inhibitor significantly suppressed HIF-2α protein expression (Figure 4A). Importantly, blocking HIF-2α resulted in a concomitant downregulation of NOX4 at both protein and transcriptional levels (Figure 4B,C), directly linking the transcription factor to this oxidative generator. Consistent with our hypothesis regarding iron transport, HIF-2α inhibition successfully reversed the intracellular Fe^2+^ accumulation (Figure 4G).

With the upstream driver blocked, we observed a comprehensive rescue of the cellular phenotype. The inhibitor treatment effectively alleviated lipid peroxidation (MDA, LPO) and reduced DCFH-DA probe fluorescence (Figure 4D–F), indicating decreased oxidative stress. Furthermore, mitochondrial function was preserved, as evidenced by the restoration of membrane potential (Figure 4H). These data establish HIF-2α as the primary driver of hyperuricemia-induced ferroptosis, acting upstream of the iron/NOX4 axis.

### 2.5. HIF-2α Knockdown Alleviates Hyperuricemia-Induced Ferroptosis in Hepatocytes

To ask whether HIF-2α’s role was genuinely causal rather than an artifact of drug treatment, we knocked down HIF-2α directly in L02 hepatocytes and asked what changed.

**Figure 5:**
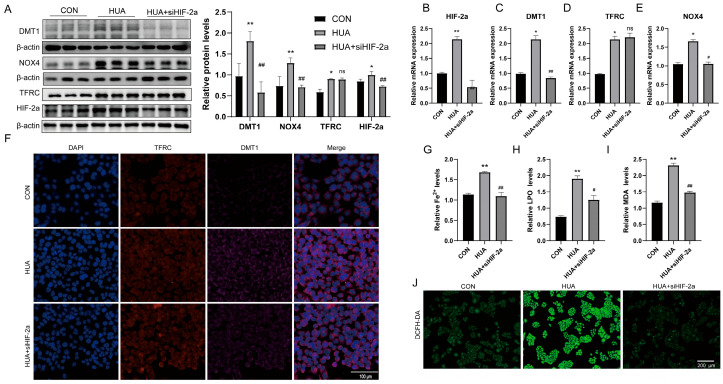
HIF-2α knockdown alleviates hyperuricemia-induced ferroptosis in hepatocytes. (**A**) Western blot analysis of TFRC, DMT1, NOX4, and HIF-2α protein expression in L02 cells after HIF-2α knockdown (*n* = 3). (**B**–**E**) HIF-2α, DMT1, TFRC, and NOX4 mRNA levels in L02 cells after HIF-2α knockdown detected by qRT-PCR (*n* = 3). (**F**) Immunofluorescence staining of DMT1 and TFRC proteins in L02 cells after HIF-2α knockdown [Immunofluorescence staining showing TFRC (red), DMT1 (purple), and nuclei counterstained with DAPI (blue), *n* = 3]. (**G**–**I**) Fe^2+^, LPO and MDA levels in L02 cells after HIF-2α knockdown (*n* = 3). (**J**) DCFH-DA probe fluorescence in L02 cells after HIF-2α knockdown (*n* = 3). * *p* < 0.05, ** *p* < 0.01 vs. control group, ^#^ *p* < 0.05, ^##^ *p* < 0.01 vs. model group, ns, not significant vs. model group.

Knockdown efficiency was confirmed at both mRNA and protein levels (Figure 5A,B and Figure S2). The downstream picture was not what we had expected in full. DMT1 and NOX4 both fell with HIF-2α (Figure 5A,C,E), but TFRC was unaffected—neither transcript nor protein moved (Figure 5A,D,F). This selective pattern was not something we had designed the experiment to test, and it raises the question of why HIF-2α appears to control DMT1 but not TFRC under these conditions.

Whether losing DMT1 alone was enough to rescue the phenotype despite intact TFRC was the next question. It was. Intracellular Fe^2+^ dropped with HIF-2α knockdown (Figure 5G), and downstream markers followed: LPO and MDA both declined (Figure 5H–I), and DCFH-DA fluorescence fell accordingly (Figure 5J), pointing to a genuine reduction in oxidative burden rather than a partial effect. Thus, HIF-2α knockdown suppressed DMT1 and NOX4 expression at both mRNA and protein levels, whereas TFRC expression remained unchanged under the same conditions.

## 3. Discussion

How hyperuricemia drives hepatic injury at the molecular level has not been clearly established, and whether ferroptosis plays a substantive role in this process was uncertain before the present study. Here we show that ferroptosis contributes meaningfully to UA-induced hepatocyte death, and that this response is regulated, at least in part, through the HIF-2α/DMT1 axis. Working through the data, UA elevated HIF-2α expression and this was accompanied by increased DMT1-mediated iron uptake, intracellular iron accumulation, NOX4 activation, lipid peroxidation, and mitochondrial impairment in sequence. We should be clear that this ordering is inferred from static intervention experiments—pharmacological blockade and genetic knockdown—rather than from real-time tracking of each transition; intermediate steps may well involve regulatory nodes we did not measure. Even so, the fact that disrupting HIF-2α by two independent methods reduced the ferroptotic phenotype consistently points to this factor as an early and rate-influencing node in the injury process. Whether it functions as the primary sensor linking UA to iron dysregulation, or whether other upstream inputs converge on the same pathway under different physiological conditions, our current data cannot resolve.

An important observation from our dose–response studies is the biphasic effect of UA on hepatocyte viability. At low concentrations (250 μM), UA exhibited minimal cytotoxicity, whereas higher concentrations (≥500 μM) induced progressive cell death. This finding reconciles the apparent paradox regarding uric acid’s biological activities. At physiological concentrations, UA functions as an antioxidant through multiple mechanisms, including iron chelation and free radical scavenging, as demonstrated by Ames [26] and Davies [27]. These studies showed that urate-Fe^3+^ complexes can inhibit iron-catalyzed lipid peroxidation in extracellular, protein-free systems. However, at pathological concentrations (≥500 μM) observed in hyperuricemia, the balance shifts decisively toward pro-oxidant effects.

Our data suggest that this transition involves HIF-2α activation and subsequent iron dysregulation. Notably, HIF-2α expression increased across all tested concentrations (Figure 2C), but the cellular consequences became detrimental only when iron accumulation exceeded homeostatic capacity. This can be explained by the fact that urate’s iron-chelating capacity, characterized by a relatively modest association constant (Ka ≈ 1.9 × 10^4^ for urate-Fe^2+^), is insufficient to overcome the sustained, active increase in intracellular iron import driven by HIF-2α-mediated DMT1 upregulation [28,29,30]. Furthermore, the iron-chelating properties of urate have been characterized predominantly in extracellular environments where urate concentrations approach saturation and free iron is extremely low—conditions fundamentally different from the intracellular labile iron pool in hepatocytes [28,31,32,33].

Additionally, at high concentrations, UA exhibits well-documented pro-oxidant properties through indirect activation of NADPH oxidase (NOX), as evidenced by our observation of NOX4 upregulation (Figure 1M,N). This NOX4-mediated superoxide generation represents an iron-independent pro-oxidant mechanism that operates in parallel with iron-driven Fenton chemistry. This concentration-dependent transition from antioxidant to pro-oxidant has important clinical implications: mild elevations in uric acid might be compensatory or even protective in certain contexts, whereas severe hyperuricemia triggers pathological cascades including ferroptosis.

Both TFRC and DMT1 were elevated in the HUA model, yet HIF-2α knockdown suppressed DMT1 while leaving TFRC largely intact. Notably, this selective loss of DMT1 was sufficient to normalize intracellular iron and abolish ferroptosis, even with TFRC still upregulated. The simplest reading of this dissociation is that HIF-2α-driven DMT1 induction is the functionally dominant route for pathological iron entry under these conditions, with TFRC playing a lesser role [34,35,36]. We cannot rule out that this balance shifts in other contexts, however. Why HIF-2α targets DMT1 and not TFRC is not something our data can answer. Differences in promoter accessibility, co-factor availability, or HRE configuration are reasonable guesses, but chromatin immunoprecipitation and promoter work would be needed to test any of them.

DMT1, initially characterized as a proton-coupled metal transporter essential for intestinal iron absorption [37], has been implicated in hepatic iron loading under pathological conditions [38]. Our data extend these findings by demonstrating that in hyperuricemic hepatocytes, DMT1 serves as the principal HIF-2α-responsive iron importer driving ferroptotic cell death. This finding has important mechanistic implications: while TFRC-mediated transferrin-bound iron uptake represents the canonical pathway in most cell types, our results suggest that under hyperuricemic stress, the DMT1-mediated non-transferrin-bound iron (NTBI) import pathway becomes the dominant route for pathological iron accumulation in hepatocytes.

We used to think UA kills hepatocytes mainly through apoptosis—our earlier papers showed this through HIF-1α upregulation and arginine pathway disruption [39], and separately through PISD downregulation [40]. Ferroptosis running in parallel was not something we expected. The Fer-1 data are fairly clear on this: the inhibitor rescued both viability and LDH release without touching apoptotic signaling, so the two deaths are happening independently, not one downstream of the other. A single stressor driving multiple death pathways at once is not unusual—Galluzzi et al. laid this out in the 2018 NCCD recommendations—but seeing it here under hyperuricemic conditions adds a layer of complexity to the picture.

Which pathway dominates, and whether that shifts with UA dose or exposure time, we cannot say from what we have. We also never tested blocking both pathways at once, so whether that would do better than targeting either alone is still open. What we can say is that just lowering uric acid, or going after caspases alone, may not be enough once ferroptosis is already running—but that needs testing before we push the point further.

From a translational perspective, current clinical management of hyperuricemia primarily focuses on reducing serum UA concentrations. However, our data suggest that once the ferroptotic cascade is initiated, urate-lowering strategies alone may be insufficient to prevent hepatic damage [41,42]. The demonstrated efficacy of both HIF-2α inhibition and iron chelation in reversing the pathological phenotype highlights potential hepatoprotective strategies that operate independently of systemic purine metabolism. Notably, selective HIF-2α antagonists have demonstrated clinical efficacy in renal cell carcinoma and are currently under investigation for other indications [43,44], suggesting potential repurposing opportunities for metabolic liver diseases.

This study has several gaps worth being direct about. We worked almost entirely in L02 cells, and primary human hepatocytes may not behave the same way. The mouse data are encouraging for HIF-2α inhibition, but we did not assess long-term safety, which limits what we can claim therapeutically. We also cannot explain why HIF-2α selectively upregulates DMT1 rather than TFRC; differential promoter accessibility or co-factor recruitment seem reasonable [45], but we did not test either directly. And whether any of this translates to hyperuricemic patients with hepatic disease, cells and mice cannot tell us. At higher UA concentrations, NOX4 protein levels rose markedly (Figure 1M,N), suggesting NADPH oxidase activation as a second oxidative source. Unlike Fenton chemistry, this route does not require labile iron, though whether the two mechanisms act independently or interact under our conditions remains unclear, as we did not directly quantify their relative contributions. The concentration-dependent shift from antioxidant to pro-oxidant behavior is notable. Whether modest uric acid elevation is genuinely cytoprotective in vivo, and at what threshold injury dominates, our data cannot determine.

Taken together, our data support a model in which UA-induced HIF-2α upregulation drives DMT1-dependent iron import, with downstream iron overload, NOX4 activation, and mitochondrial damage converging on ferroptosis as the cell death outcome. HIF-2α inhibition reduced this phenotype consistently across our experimental systems, which makes the HIF-2α/DMT1 axis worth pursuing as a therapeutic target—though how well this translates beyond cell and mouse models remains to be seen.

## 4. Materials and Methods

### 4.1. Reagents and Antibodies

Uric acid (UA, Cat# U2625), sodium hydroxide (NaOH, Cat# S8045), glycine (Cat# G7126), and dimethyl sulfoxide (DMSO, Cat# D2650) were purchased from Sigma-Aldrich (St. Louis, MO, USA). Deferoxamine (DFO, Cat# HY-B0988) and HIF-2α-IN-4 (Cat# HY-125840) were obtained from MedChemExpress (Monmouth Junction, NJ, USA). RPMI-1640 medium (Cat# SH30027.01) was purchased from Hyclone (Logan, UT, USA). Fetal bovine serum (FBS-BE500) was obtained from NEWZERUM (Shanghai, China). Cell culture flasks (Cat# 430641), 6-well plates (Cat# 3516), and cryovials (Cat# 430488) were purchased from Corning (Corning, NY, USA).

The BCA protein assay kit (Cat# P0012S), ROS assay kit (DCFH-DA, Cat# S0033S), JC-1 mitochondrial membrane potential assay kit (Cat# C2006), MDA assay kit (Cat# S0131S), and TRIzol RNA extraction kit (Cat# R0016) were purchased from Beyotime Biotechnology (Shanghai, China). UA assay kit (Cat# C012-2-1), AST assay kit (Cat# C010-2-1), and ALT assay kit (Cat# C009-2-1) were obtained from Nanjing Jiancheng Bioengineering Institute (Nanjing, China). Fe^2+^ assay kit (Cat# E-BC-K773-M) and LPO assay kit (Cat# E-BC-K176-M) were purchased from Elabscience (Wuhan, China). FerroOrange probe (Cat# F374) was obtained from Dojindo Laboratories (Kumamoto, Japan). SYBR Green qRT-PCR Master Mix (Cat# GT131) was purchased from Greact (Beijing, China).

Protein marker (Cat# 26616) was obtained from Thermo Fisher Scientific (Waltham, MA, USA). ECL chemiluminescence substrate (Cat# 1705061) was purchased from Bio-Rad (Hercules, CA, USA). PVDF membranes (Cat# IPVH00010) were obtained from Merck Millipore (Darmstadt, Germany). Anti-HIF-2α antibody (Cat# ET7107-32)and anti-NOX4 antibody (Cat# EM1706-66) were obtained from HuaBio (Hangzhou, China). Anti-DMT1 antibody (Cat# YP-mAb-08042) and anti-TFRC antibody (Cat# YP-Ab-14016) were purchased from UpingBio (Guangzhou, China). Anti-β-actin antibody (Cat# AF7018) was obtained from Affinity Biosciences (Cincinnati, OH, USA).

### 4.2. Animals and Hyperuricemia Model

Six-week-old male C57BL/6J mice (body weight 18–22 g) were obtained from Hangzhou Ziyuan Laboratory Animal Technology Co., Ltd. (Hangzhou, China). A total of 12 mice were used in this study. Mice were housed in clear plastic cages (five per cage) under specific pathogen-free (SPF) conditions at a controlled temperature of 22 ± 2 °C and relative humidity of 50 ± 10%, with a 12 h light/dark cycle, and had ad libitum access to standard rodent diet and water provided by Hangzhou Ziyuan Laboratory Animal Technology Co., Ltd. (Hangzhou, China). Animals were acclimatized for one week prior to the start of experiments.

Mice were randomly divided into two groups: the control group and the hyperuricemia (HUA) model group (*n* = 6 per group). The control group was fed a standard chow diet, while the HUA model group was fed a specialized diet containing 2% potassium oxonate and 3% UA for 8 weeks. After model establishment, mice were anesthetized with isoflurane, and blood samples were collected via retro-orbital bleeding for serum biochemical analyses, including UA, AST, and ALT levels. Mice were then euthanized by gradual-fill carbon dioxide (CO_2_, gradually increased to 80%). Liver tissues were subsequently harvested for histopathological examination (H&E staining), transmission electron microscopy, Western blot analysis, qRT-PCR, and biochemical assays (MDA, LPO, and Fe^2+^ quantification).

### 4.3. Cell Culture and Treatment

The human hepatocyte cell line L02 was obtained from the Cell Bank of the Chinese Academy of Sciences (Shanghai, China). Cells were cultured in RPMI-1640 medium supplemented with 10% FBS and 1% penicillin-streptomycin at 37 °C in a humidified atmosphere containing 5% CO_2_.

UA stock solution (50 mM) was prepared by dissolving 0.0252 g UA powder in 3 mL of 1 M NaOH solution, adjusting pH to 7.2–7.4 with concentrated HCl, and filtering through a 0.22 μm membrane. For UA treatment, L02 cells were exposed to UA (750 μM) for 48 h. For pharmacological intervention, cells were pretreated with DFO (100 μM) or HIF-2α-IN-4 (10 μM) for 2 h prior to UA exposure. Note: To exclude the possibility that observed effects were mediated by monosodium urate (MSU) crystal formation rather than soluble uric acid, we performed microscopic examination under polarized light. No birefringent crystals were detected in either culture medium or cell lysates at any of the UA concentrations tested (250–1000 μM), confirming that all experimental effects were attributable to soluble UA rather than crystal-induced inflammation (Supplementary Figure S3).

### 4.4. Cell Viability and Cytotoxicity Assays

Cell viability (CCK-8 assay): L02 cells were seeded in 96-well plates at a density of 5 × 10^3^ cells/well and treated with UA at various concentrations (0, 250, 500, 750, 1000 μM) for 48 h. CCK-8 reagent (10 μL) was added to each well and incubated for 2 h at 37 °C. Absorbance was measured at 450 nm using a microplate reader. Cell viability was calculated as a percentage of the control group.

L02 cells were seeded in 96-well plates at an appropriate density (1× 10^4^ cells/well) to ensure confluence did not exceed 60–70% at the time of detection. Prior to treatment, culture medium was removed, cells were washed once with PBS, and replaced with low-serum medium (containing 1% FBS). Wells were designated as follows: blank control wells (medium only, no cells), sample control wells (untreated cells), maximum LDH activity control wells (untreated cells for subsequent lysis), and drug-treated wells (UA-treated cells). At 1 h before the designated detection time point, LDH release reagent provided in the kit was added to the maximum LDH activity control wells at 10% of the original culture volume, mixed thoroughly by pipetting, and cells were returned to the incubator. At the designated time point, plates were centrifuged at 400× *g* for 5 min. Supernatants (120 μL) from each well were transferred to a new 96-well plate for LDH activity measurement using a commercial LDH cytotoxicity assay kit (Beyotime Biotechnology, Cat# C0016, Shanghai, China) according to the manufacturer’s instructions. LDH release was calculated as a percentage of total LDH (supernatant + cell lysate) and normalized to the control group. NOTE: At this seeding density (1 × 10^4^ cells/well), the spontaneous LDH release in vehicle-treated control cells was approximately 0.5 U/mL, which is consistent with the low-concentration detection range of the assay kit (standard curve range: 15.6–250 mU/mL), confirming that the baseline signal reflects the intrinsic LDH content of the cell number used rather than a methodological limitation.

Ferrostatin-1 rescue experiment: L02 cells were pretreated with Ferrostatin-1 (Fer-1, 1 μM; MedChemExpress, Cat# HY-100579) for 2 h prior to UA exposure (750 μM, 48 h). Cell viability and LDH release were then assessed as described above.

### 4.5. Serum Biochemical Analysis

Serum UA levels were measured using a commercial UA assay kit (Nanjing Jiancheng Bioengineering Institute, Cat# C012-2-1, Nanjing, China) based on the phosphotungstic acid reduction method. Briefly, serum samples were mixed with tungstate protein precipitant and centrifuged at 1500× *g* for 5 min. The supernatant was collected and reacted with CUT reagent and phosphotungstic acid reagent at room temperature for 10 min. Absorbance was measured at 600 nm using a microplate reader (Thermo Fisher Scientific), and UA concentrations were calculated against a standard curve.

Serum ALT levels were determined using a commercial ALT assay kit (Nanjing Jiancheng Bioengineering Institute, Cat# C009-2-1, Nanjing, China) based on the Reitman-Frankel colorimetric method. Briefly, substrate solution (pre-warmed at 37 °C) was incubated with serum samples at 37 °C for 30 min, followed by addition of 2,4-dinitrophenylhydrazine (DNPH) solution and further incubation at 37 °C for 20 min. After addition of 0.4 mol/L NaOH solution, the mixture was incubated at room temperature for 15 min. Absorbance was measured at 505 nm, and enzyme activities were calculated according to a standard curve and expressed.

Serum AST levels were determined using a commercial AST assay kit (Nanjing Jiancheng Bioengineering Institute, Cat# C010-2-1, Nanjing, China) based on the Reitman-Frankel colorimetric method. Briefly, substrate solution (pre-warmed at 37 °C) was incubated with serum samples at 37 °C for 30 min, followed by addition of DNPH solution and further incubation at 37 °C for 20 min. After addition of 0.4 mol/L NaOH solution, the mixture was incubated at room temperature for 15 min. Absorbance was measured at 510 nm, and enzyme activities were calculated according to a standard curve and expressed.

### 4.6. Histopathological Examination

Liver tissues were fixed in 4% paraformaldehyde for 48 h, dehydrated, and embedded in paraffin. Sections (4 μm) were cut using a microtome (RM2255, Leica Biosystems, Wetzlar, Germany) and stained with hematoxylin and eosin (H&E) according to standard protocols. Briefly, sections were deparaffinized in xylene (2 × 20 min), rehydrated through graded ethanol (100% for 2 × 10 min, 90%, 80%, 70% for 5 min each), stained with hematoxylin for 5 min, rinsed under running water for 5 min, stained with eosin for 15 s, dehydrated through graded ethanol and xylene, and mounted with neutral resin. Images were captured using a slide scanner (Pannoramic MIDI, 3DHISTECH Ltd., Budapest, Hungary).

### 4.7. Transmission Electron Microscopy

For ultrastructural analysis, fresh liver tissues were cut into 1 mm^3^ pieces on ice and fixed in 3% glutaraldehyde containing 2% paraformaldehyde at 4 °C. After washing with PBS (3 × 15 min), samples were post-fixed in 2% osmium tetroxide for 1 h, followed by PBS washing (3 × 15 min). Samples were dehydrated through graded ethanol (50%, 70%, 90% for 15 min each) and acetone (100%, 2 × 20 min), infiltrated with acetone/resin mixture (1:1) for 2 h at room temperature, and embedded in pure resin overnight. After polymerization at 37 °C for 24 h and 60 °C for 48 h, ultrathin sections (60 nm) were cut using an ultramicrotome, stained with uranyl acetate and lead citrate, and examined under a transmission electron microscope (Talos L120C, Thermo Fisher Scientific, Waltham, MA, USA) operated at 120 kV. Mitochondrial morphology was assessed for characteristic ferroptotic features, including mitochondrial shrinkage, increased membrane density, and cristae reduction.

### 4.8. Western Blot Analysis

Total protein was extracted from liver tissues or L02 cells using RIPA lysis buffer supplemented with protease inhibitors. Protein concentrations were determined using a BCA protein assay kit (Beyotime Biotechnology, Cat# P0012S, Shanghai, China). Equal amounts of protein (30–50 μg) were separated by 10% SDS-PAGE and transferred onto PVDF membranes (Merck Millipore, Darmstadt, Germany). Membranes were blocked with 5% skim milk in TBST for 1 h at room temperature and incubated with primary antibodies against HIF-2α (HuaBio, Cat# ET7107-32, Hangzhou, China), NOX4 (HuaBio, Cat# EM1706-66, Hangzhou, China), DMT1 (UpingBio, Cat# YP-mAb-08042, Guangzhou, China), TFRC (UpingBio, Cat# YP-Ab-14016, Guangzhou, China), or β-actin (Affinity Biosciences, Cat# AF7018, Cincinnati, OH, USA) at 4 °C overnight. After washing with TBST, membranes were incubated with HRP-conjugated secondary antibodies for 1 h at room temperature. Protein bands were visualized using an ECL detection system (Bio-Rad Laboratories, Cat# 1705061, Hercules, CA, USA) and captured using a ChemiDoc MP Imaging System (Version 2.4, Bio-Rad Laboratories, Hercules, CA, USA). Band intensities were quantified using ImageJ 1.53q software (National Institutes of Health, Bethesda, MD, USA) [46].

### 4.9. Quantitative Real-Time PCR

Total RNA was extracted from liver tissues (50 mg) or L02 cells using TRIzol reagent following the manufacturer’s protocol. RNA concentration and purity were assessed using a micro-spectrophotometer (Thermo Fisher Scientific, Waltham, MA, USA). Complementary DNA (cDNA) was synthesized from 1 μg of total RNA using a reverse transcription kit under the following conditions: 37 °C for 15 min, 85 °C for 5 s. qRT-PCR was performed using SYBR Green Master Mix on a real-time PCR system with the following program: 95 °C for 30 s, followed by 40 cycles of 95 °C for 5 s and 60 °C for 30 s. Relative mRNA expression levels were calculated using the 2^−ΔΔCt^ method and normalized to β-actin. Primer sequences are listed in Table S1.

### 4.10. Detection of Oxidative Stress and Iron Markers

MDA and LPO: MDA levels in liver tissues or L02 cells were measured using a commercial MDA assay kit. LPO levels were determined using an LPO assay kit. Results were normalized to protein concentration.

DCFH-DA probe fluorescence: Intracellular oxidant levels were detected using the DCFH-DA fluorescent probe (Cat#S0033S; Beyotime Biotechnology, Shanghai, China). L02 cells were incubated with DCFH-DA (10 μM) at 37 °C for 30 min in the dark. After washing with serum-free medium, fluorescence was observed under a laser confocal microscope or high-content imaging system. IMPORTANT NOTE: DCFH-DA can be oxidized directly by Fe^2+^ via 1-electron reactions and therefore reports any 1-electron oxidant including iron and copper, not exclusively superoxide or other non-metal ROS Therefore, in experiments involving iron manipulation, DCF fluorescence may partly reflect iron-mediated oxidation. Oxidative stress conclusions are supported by multiple iron-independent markers including MDA, LPO, NOX4, and GPX4.

Intracellular Fe^2+^: Intracellular ferrous iron levels were measured using the FerroOrange probe. Cells were incubated with FerroOrange working solution (1 μM) at 37 °C for 30 min in the dark, and fluorescence images were captured using a confocal microscope. Fluorescence intensity was quantified using ImageJ software. For liver tissues, Fe^2+^ content was determined using an iron assay kit following the manufacturer’s protocol.

Mitochondrial membrane potential (MMP): MMP was assessed using the JC-1 assay kit. Cells were incubated with JC-1 working solution at 37 °C for 20 min. After washing with JC-1 staining buffer, cells were observed under a fluorescence microscope. In healthy cells with high MMP, JC-1 aggregates in mitochondria and emits red fluorescence. In cells with low MMP, JC-1 remains as monomers in the cytoplasm and emits green fluorescence. The ratio of red to green fluorescence intensity was calculated as an indicator of MMP.

### 4.11. Statistical Analysis

All data are presented as mean ± standard deviation (SD) from at least three independent experiments. Statistical analyses were performed using GraphPad Prism 9.0 software (GraphPad Software, San Diego, CA, USA). Differences between two groups were analyzed using unpaired Student’s *t*-test. Comparisons among multiple groups were performed using one-way analysis of variance (ANOVA) followed by Tukey’s post hoc test. A *p* value < 0.05 was considered statistically significant. * *p* < 0.05, ** *p* < 0.01 versus control group; ^#^ *p* < 0.05, ^##^ *p* < 0.01 versus HUA group.

## Data Availability

The original contributions presented in this study are included in the article/Supplementary Materials. Further inquiries can be directed to the corresponding authors.

## Supplementary Materials

The following supporting information can be downloaded at: https://www.mdpi.com/article/10.3390/ijms27062833/s1.

## Abbreviations

The following abbreviations are used in this manuscript:

HUA	High Uric Acid
HUM	Hyperuricemia Mouse Model
UA	Uric Acid
AST	Aspartate Aminotransferase
ALT	Alanine Aminotransferase
TEM	Transmission ElectronMicroscopy
NOX4	NADPHOxidase4
ROS	ReactiveOxygenSpecies
Fe^2+^	FerrousIon
HIF-2α	Hypoxia-InducibleFactor2α
mRNA	Messenger RNA
DMT1	DivalentMetalTransporter1
TFRC	TransferrinReceptor
LPO	Lipid peroxidation
MDA	Malondialdehyde
MMP	MitochondrialMembranePotential
qRT-PCR	QuantitativePolymeraseChainReaction
TF	Transferrin
STEAP3	Six-TransmembraneEpithelialAntigenofProstate3
LIP	LabileIronPool
HIF	Hypoxia-InducibleFactor
PHDs	ProlylHydroxylases
VHL	vonHippel-Lindau
HREs	HypoxiaResponseElements
EPO	Erythropoietin
DFO	Deferoxamine
Fer-1	Ferrostatin-1
LDH	Lactate dehydrogenase
CCK-8	Cell Counting Kit-8
