# Supplementary material for: Uric Acid Induces Hepatocytes Ferroptosis Through HIF-2α/DMT1-Mediated Iron Overload
Source: Int J Mol Sci. 2026 Mar 20;27(6):2833. doi: 10.3390/ijms27062833 (PMC13026954; doi:10.3390/ijms27062833)

Uric acid induces hepatocytes ferroptosis through HIF-2α-mediated iron overload

Tao Wang [1], Wanbao Zheng [1], Meimei Guo [2], Jun Cao [1], Li Wang [2], Marco Sim Kah How [3], Youzhi Xu [2], * and Wenjie Lu [1], *

[1] School of Pharmacy , Anhui Medical University, Hefei, Anhui, 230032, China

[2] Basic Medical College, Anhui Medical University, Hefei, Anhui, 230032, China

[3] International College, Anhui Medical University, Hefei, Anhui, 230032, China

***** Correspondence: Wenjie Lu (wenjielu@ahmu.edu.cn, ORCID: 0000-0003-1741-2311, Tel: +86-551-65161133) and Youzhi Xu (xuyouzhi@ahmu.edu.cn, ORCID: 0000-0003-2160-7576, Tel: +86-551-65161129).

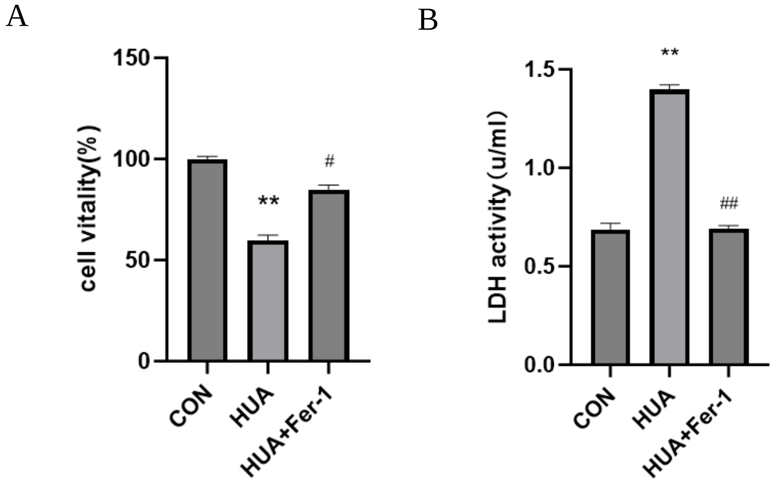

Figure S1: Ferrostatin-1 rescue experiments. (A) Fer-1 significantly rescued UA-induced cell viability loss (CCK-8 assay). (B) Fer-1 markedly attenuated UA-induced LDH release, confirming ferroptosis-specific cell death;

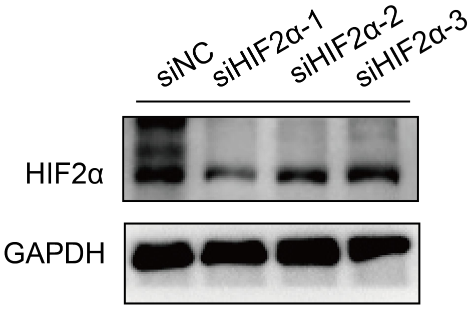

Figure S2:Knockdown efficiency of HIF-2α

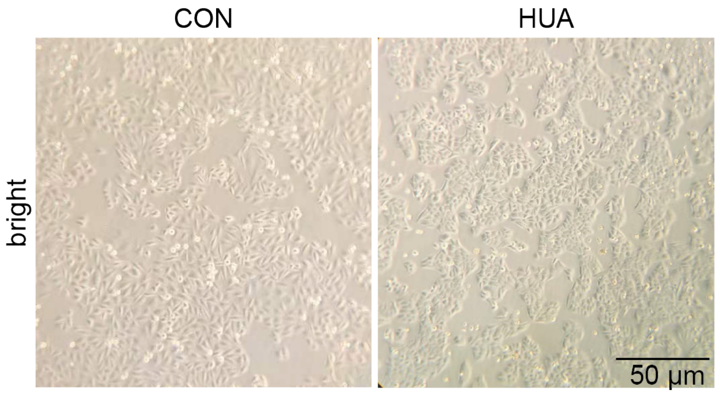

Figure S3: Absence of monosodium urate crystal formation under experimental conditions(750 μM UA, 2h)

Table S1:Primer and siRNA Sequences

	Primer F	Primer R
	Mouse	
HIF-2α	GAGAACCTGACTCTCAAAAACG	GTTGTTGTAGACTCTCACTTGC
DMT1	CCTGTGGCTGATGGTGGAGTTG	GGAGATTGATGGCGATGGCTGAG
TFRC	TCGTGGAGACTACTTCCGTGCTAC	TCTTGGAGATACATAGGGCGACAGG
	Human	
HIF-2α	GCTAGACTCCGAGAACATGACCAAG	CAGGTTGCGAGGGTTGTAGATGAC
NOX4	CAGGAGG GCTG CTGAAG-TATCAA	TGACTGGCTTATTGCTC-CGGATA
DMT1	CATCCTCACATTTACGAGCTTG	CCAACCCAAGTAGAACACAAAG
TFRC	TGAACCAATACAGAGCAGACAT	GTTTTCTCAGCATTCCCGAAAT

**siRNA Target**	**Sense Sequence (5'→3')**	**Antisense Sequence (5'→3')**
HIF-2α	GCGACAGACUGUUGGCUAUTT	AUAGCCAACAGUCUGUCGCTT
Negative Control	UUCUCCGAACGUGUCACGUTT	ACGUGACACGUUCGGAGAATT

Figure S4：Original Western blot images

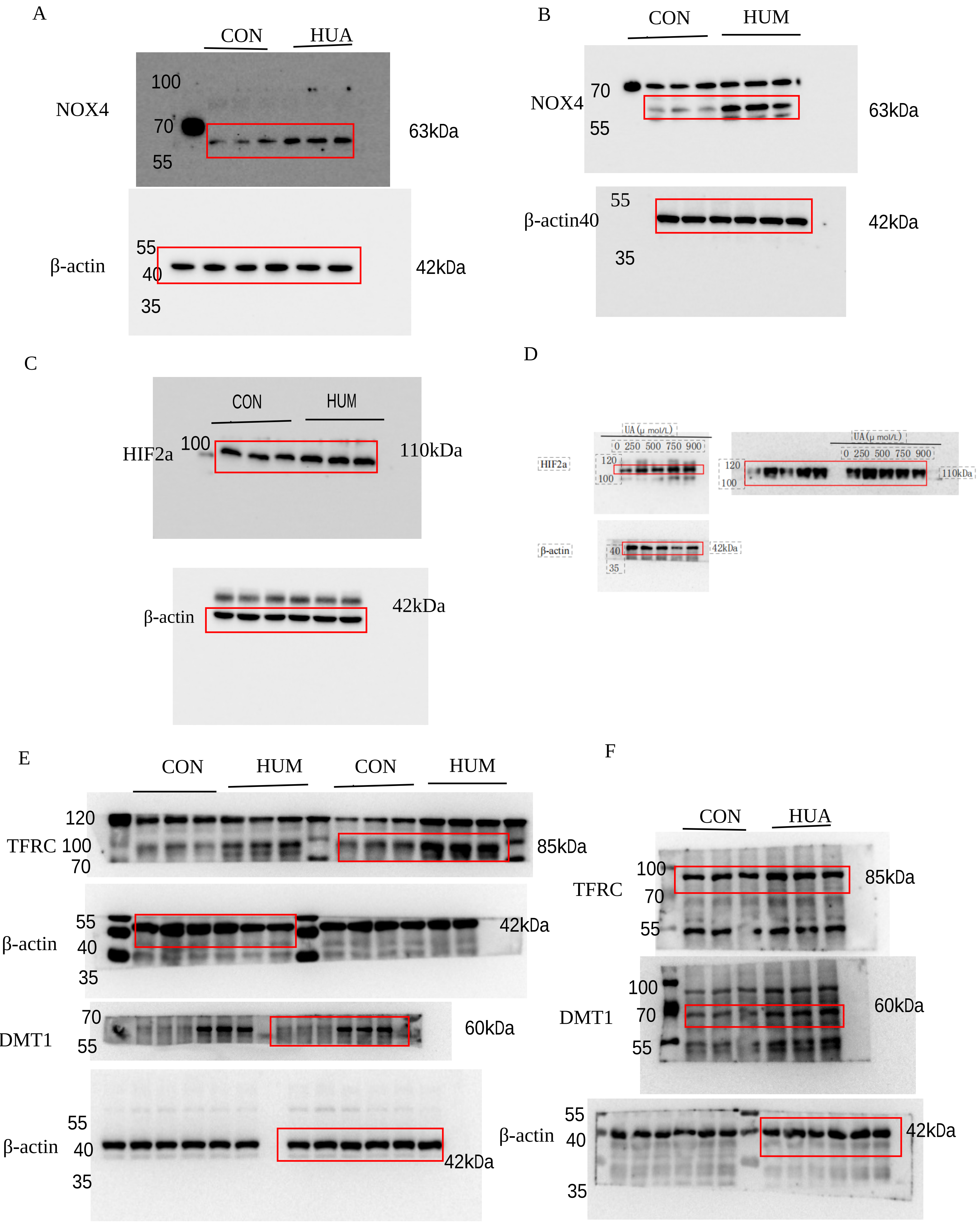

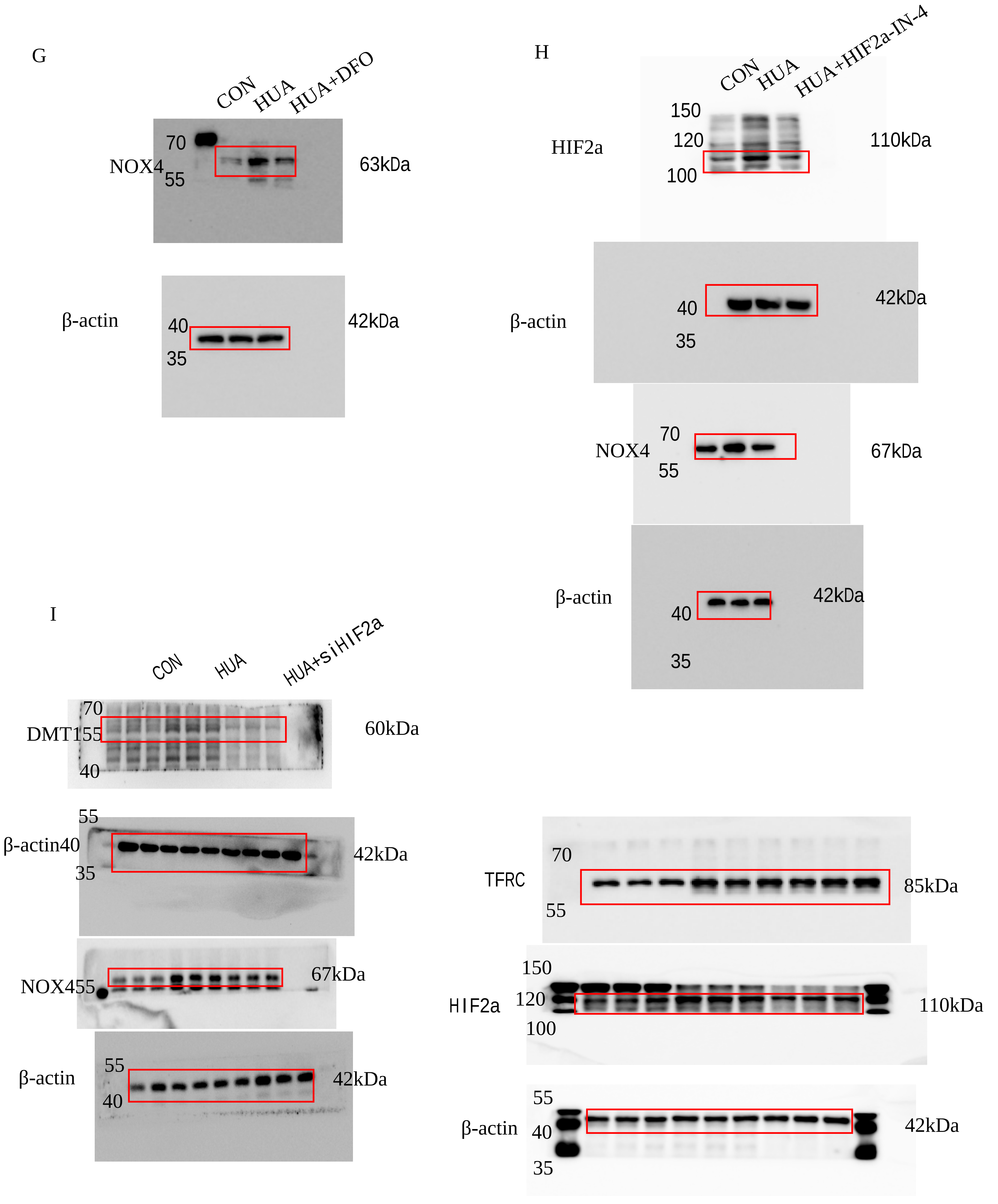
G
CON
HUA
HUA+DFO
NOX4
70
55
63kDa
β-actin
40
35
42kDa
H
CON
HUA
HUA+HIF2a-IN-4
HIF2a
150
120
100
110kDa
β-actin
40
35
42kDa
NOX4
70
55
67kDa
β-actin
40
35
42kDa
I
CON
HUA
HUA+siHIF2a
DMT1
70
55
40
60kDa
β-actin
55
40
35
42kDa
NOX4
55
67kDa
β-actin
55
40
42kDa
TFRC
70
55
85kDa
HIF2a
150
120
100
110kDa
β-actin
55
40
35
42kDa

J

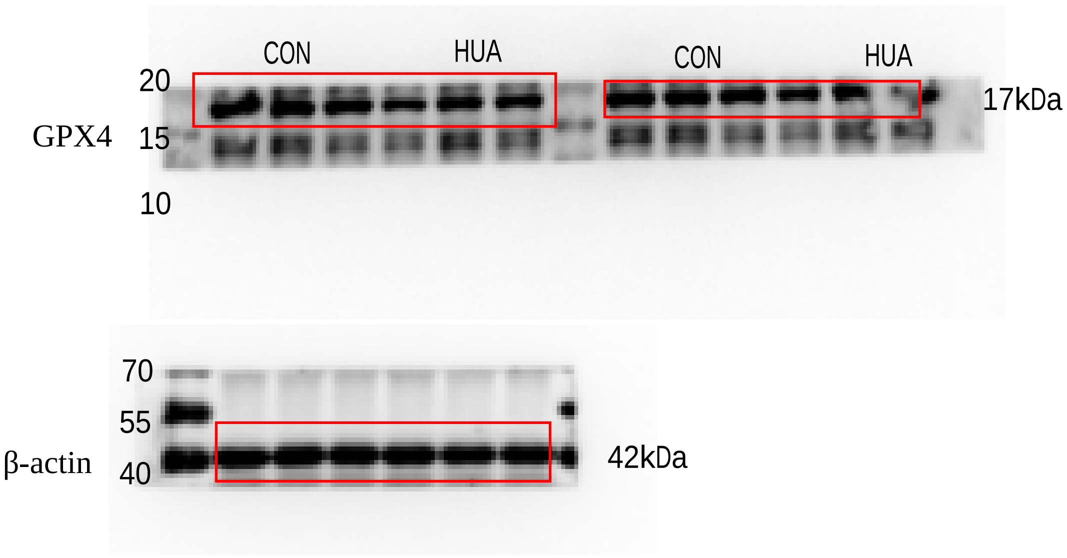

Supplement: Supplementary file 1 [file ijms-27-02833-s001.zip › ijms-4187862-supplementary.pdf]
